# Global MyoG research 2004–2024: a bibliometric analysis of trends and translational implications

**DOI:** 10.3389/ebm.2026.10929

**Published:** 2026-03-05

**Authors:** Luoming Hu, Weizhong Zhuang, Weimin Chen, Song Yang, Shuo Chen, Xin Wang, Qiang Gao, Jimei Chen

**Affiliations:** 1 Department of Cardiac Surgery, Guangdong Provincial People’s Hospital, Guangdong Academy of Medical Sciences, Southern Medical University, Guangzhou, China; 2 Guangdong Cardiovascular Institute, Guangdong Provincial People’s Hospital, Guangdong Academy of Medical Sciences, Southern Medical University, Guangzhou, China; 3 Guangdong Provincial Key Laboratory of South China Structural Heart Disease, Guangzhou, China; 4 School of Medicine South China University of Technology, Guangzhou, China

**Keywords:** bibliometric analysis, muscle atrophy, myogenin (MyoG), regenerative medicine, rhabdomyosarcoma

## Abstract

Myogenin (MyoG) is a core myogenic transcription factor that orchestrates myoblast differentiation and myofiber maturation and has been increasingly implicated in skeletal muscle degeneration and rhabdomyosarcoma, yet its global research landscape has not been systematically characterized. In this study, we performed a bibliometric analysis of MyoG-related publications from 2004 to 2024 retrieved from the Web of Science Core Collection. A total of 402 articles authored by 2,402 researchers from 1,148 institutions across 165 countries and regions were analyzed using VOSviewer, CiteSpace and R-based bibliometric tools. We quantified annual publication output, identified leading countries, institutions, authors and journals, and reconstructed collaboration, co-citation and keyword co-occurrence networks to delineate thematic evolution. The global pattern showed a multipolar structure dominated by the United States and China, with European institutions forming an additional hub and emerging countries contributing with growing but comparatively lower impact. Research hotspots exhibited a clear progression from early work on molecular mechanisms (DNA binding, MyoD family interactions, chromatin remodelling) toward regenerative biology (satellite cell regulation, muscle regeneration) and, more recently, disease-oriented studies focused on muscle atrophy, Duchenne muscular dystrophy and rhabdomyosarcoma. Landmark co-cited studies established MyoG as an indispensable regulator of skeletal muscle differentiation and highlighted its expanding relevance in pathological remodelling and therapeutic targeting. Future work is expected to concentrate on decoding MyoG-centred regulatory networks in degenerative muscle disease, integrating single-cell and spatial transcriptomics with functional genomics and multi-omics, and developing MyoG-based diagnostic and targeted therapeutic strategies. Despite the intrinsic limitations of single-database and citation-based approaches, this study provides a panoramic overview of two decades of MyoG research and offers a structured framework to guide future basic and translational investigations in muscle biology and oncology.

## Impact statement

Myogenin is a key gene that controls how muscle cells develop, repair damage, and waste away in disease, yet research on this gene is scattered across many specialties. Our study is the first to draw a clear global picture of two decades of work on myogenin, covering who is doing the research, what topics they focus on, and how interests have shifted over time. We show how the field has moved from basic muscle development toward muscle wasting in aging, inherited muscle disorders, and childhood muscle cancer. By highlighting the most influential articles, the main research teams, and the major gaps in current knowledge, this work gives experimental and clinical researchers a practical roadmap for choosing important questions, building collaborations, and designing future studies that can more directly benefit patients with muscle disease.

## Introduction

In 1987, Davis, R.L., H. Weintraub, and A.B. Lassar made a landmark discovery by converting mouse embryonic fibroblasts (C3H10T1/2 cells) into myoblasts through the expression of a single exogenous cDNA, which they subsequently named MyoD (myoblast determination gene 1) [1]. In 1989, myogenin (MyoG) was identified as a pivotal regulator of skeletal muscle differentiation. MyoG promotes myogenic differentiation and maturation through multiple mechanisms, including the activation of muscle-specific gene expression, modulation of the cell cycle, and synergistic interactions with MyoD and MRF4 [2]. As a key transcription factor in myogenesis, MyoG primarily governs myoblast differentiation and myofiber maturation. During embryonic development, MyoG drives the transition of muscle progenitor cells from the proliferative phase to the differentiated state by inducing cell-cycle exit and activating muscle-specific gene programs, thereby facilitating myofiber formation and muscle tissue maturation [3]. In injury-induced regeneration, MyoG activates the differentiation program of satellite cells, promoting their withdrawal from the cell cycle and subsequent fusion into newly formed myofibers, ultimately supporting muscle repair and functional recovery [4].

Although numerous studies have investigated myogenin (MyoG) in recent years, a comprehensive and systematic literature synthesis is still lacking. Bibliometrics is a quantitative approach that integrates theories and methodologies from statistics, mathematics, and related disciplines. By visualizing and analyzing scientific publications, it effectively presents large-scale literature data in structured knowledge forms, enabling researchers to rapidly identify current trends, key pathways and nodes, research hotspots, and advances within their fields. In addition, bibliometrics helps optimize the allocation of existing research resources to address ongoing scientific challenges. Therefore, this study employs bibliometric methods to systematically summarize MyoG-related publications from 2004 to 2024, further analyzing shifts in research focus, future developmental trajectories, emerging hotspots, and frontier concepts, thereby providing important guidance for future research directions and translational potential pertaining to MyoG.

## Materials and methods

### Data sources and search strategy

All bibliographic records were retrieved from the Web of Science Core Collection (WoSCC;^1^) via the institutional subscription of Southern Medical University. The final search was performed on 14 February 2025 and covered the period from 1 January 2004 to 31 December 2024. The topic search strategy was defined as TS = (myogenin OR MYOG) AND TS = (gene OR “transcription factor”).

To obtain a clearly defined and reproducible dataset, we applied explicit inclusion criteria. Eligible records were: (1) document types indexed as “Article”, “Proceedings Paper” or “Early Access”; (2) publications dated between 1 January 2004 and 31 December 2024; (3) publications written in English; and (4) studies in which myogenin (MyoG) or closely related synonyms (e.g., myogenic factor 4, myogenic regulatory factor) constituted a primary focus of the research, as reflected in the title, abstract or author keywords.

Records were excluded if they: (1) were indexed as reviews, letters, editorials, bulletins, book reviews, meeting abstracts or news items; (2) mentioned myogenin only peripherally or in a non-biological context (e.g., as part of a long gene list without specific analysis or interpretation related to myogenin); (3) were flagged as retracted in WoSCC or by journal notices; or (4) lacked essential bibliographic information (e.g., publication year or source title) required for reliable citation and network analyses.

**FIGURE 1:**
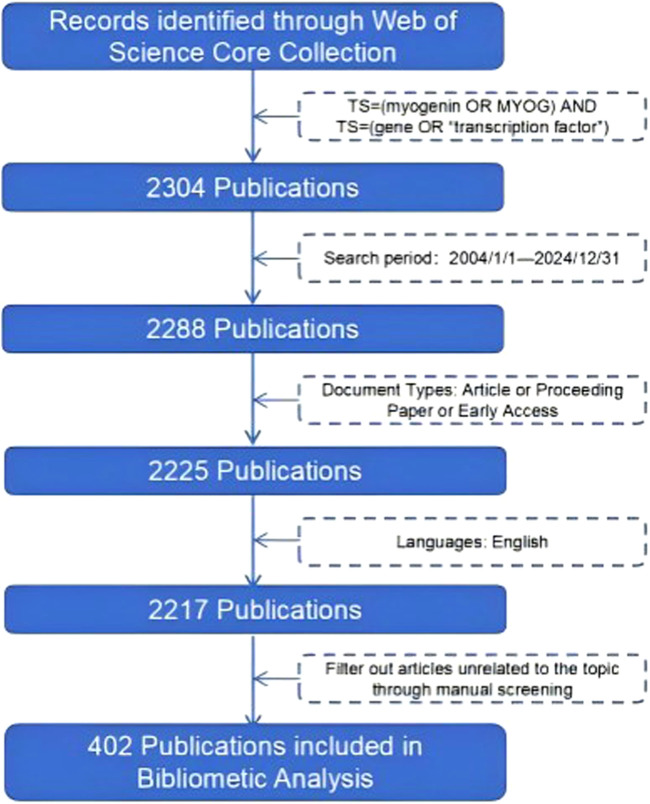
Flowchart of the literature screening process in MyoG research.

All records retrieved from WoSCC were downloaded in plain-text format with full bibliographic and citation information and imported into EndNote (Clarivate Analytics) for reference management. Potential duplicate records arising from overlapping indexes within WoSCC were identified using EndNote’s automatic duplicate detection (matching on title, first author and year) and then manually verified by checking digital object identifiers (DOIs) and journal information. Confirmed duplicates were removed, retaining a single record with the most complete metadata. Two authors independently screened the titles and abstracts of the remaining records against the inclusion and exclusion criteria, with disagreements resolved by discussion and consensus. The overall process of identification, screening, eligibility assessment and final inclusion is presented in a PRISMA-style flow diagram (Figure 1).

### Keyword selection and validation

The development of the search strategy and analytical keywords followed an iterative, structured process. First, we identified core conceptual terms related to myogenin by consulting controlled vocabularies and indexing systems, including Medical Subject Headings (MeSH) in PubMed and Keywords Plus in the Web of Science Core Collection. Candidate terms (e.g., “myogenin”, “MyoG”, “myogenic factor 4”, “myogenic regulatory factor”) were initially generated from these sources and from author keywords of seminal myogenin papers.

Second, we conducted pilot topic searches in WoSCC using different combinations of these terms and inspected the retrieved records to assess sensitivity and specificity. The final search string was refined through several iterations until additional test searches in recent years did not yield new myogenin-focused records. The approved search query was then applied consistently for the full 2004–2024 timespan.

For the keyword co-occurrence analyses, we extracted both author keywords (DE field) and Keywords Plus (ID field) from the WoSCC export files. Before analysis, two authors independently reviewed the raw keyword list to standardize terms. Standardization procedures included lowercasing, merging obvious synonyms and spelling variants (e.g., “myogenin”, “MyoG”, “myogenic factor 4”), unifying singular and plural forms, and removing overly generic methodological terms (e.g., “expression”, “protein”, “model”). Discrepancies were resolved by discussion and consensus. This keyword development and cleaning procedure was adapted from established bibliometric practices used in recent conceptual mapping studies [5, 6].

### Data analysis tools

This study employed the following tools for data processing, visualization, and statistical analysis:

1. VOSviewer (version 1.6.20): Used to construct co-occurrence networks of countries/regions, institutions, journals, and gene-related terms, thereby identifying collaboration patterns and knowledge clusters.
2. CiteSpace (version 6.2.R4): Applied for systematic analyses of national contributions, institutional collaboration networks, author co-occurrence relationships, journal co-citation networks, and keyword evolution trends, revealing the spatiotemporal dynamics of MyoG research.
3. R-based toolkits:
  - Bibliometrix: Extracted bibliometric metadata and performed basic statistical analyses;
  - ComplexHeatmap and ClusterProfiler: Visualized hierarchical clustering of research themes and conducted functional enrichment analyses.
4. OriginPro (version 2024b): Utilized for geospatial mapping and advanced data visualization, with a focus on depicting the global distribution of research outputs.
5. Pajek and Scimago Graphica: Served to refine network topology and enhance the visual presentation of collaboration networks.

For all network-based analyses, parameter settings were chosen to balance analytical stability and interpretability, in line with practices in comparable bibliometric studies. In VOSviewer, co-authorship, co-citation and keyword co-occurrence maps were constructed using association-strength normalization and the default clustering algorithm. For keyword co-occurrence, we applied a minimum occurrence threshold of 5 to exclude very infrequent terms while retaining the main thematic structure of the field. For journal and reference co-citation analyses, we used a minimum citation threshold of 20 citations. In CiteSpace, we adopted one-year time slices, cosine similarity as the link strength measure and the default clustering parameters. These parameter choices are consistent with those reported in recent bibliometric analyses of biomedical topics, ensuring comparability and robustness of the identified clusters.

## Results

### Basic quantitative information

This study integrated the contributions of 2,402 researchers from 165 countries and regions worldwide, ultimately including 402 academic publications. These studies were produced through collaborations among 1,148 research institutions and published across 220 scientific journals. In total, the research network accumulated 12,562 citations, reflecting a broad pattern of scientific collaboration spanning multiple regions and institutions.

### Annual publications trend

**FIGURE 2:**
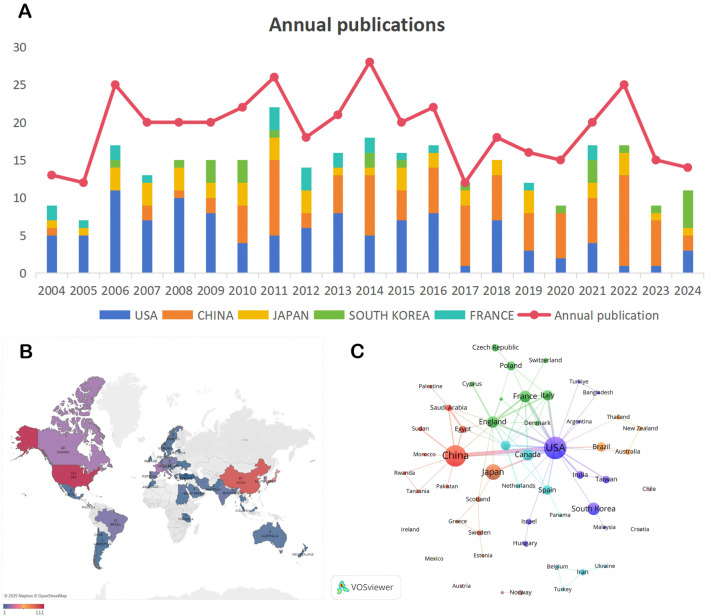
Visualizations of publication trends and international collaboration in MyoG research. **(A)** Annual publication trends in the MyoG field from 2004 to 2024, accompanied by a bar chart of the top five contributing countries, highlighting overall research activity and leading contributors. **(B)** Geographic distribution of total publications by country, illustrating global research output in the MyoG domain. **(C)** International collaboration network, depicting research partnerships and contributions among countries, with emphasis on collaborative linkages and scientific influence.

This study tracked the annual publication output of MyoG-related literature from 2004 to 2024. As shown in Figure 2A, the number of publications rose from 13 in 2004 to a peak of 25 in 2006. Although a decline followed, the annual output soon stabilized at approximately 20 publications, indicating rapid progress in MyoG research during the 2004–2006 period. Another notable peak appeared between 2011 and 2014, with an average of 23 publications per year. A subsequent rise occurred in 2022, reaching 25 publications. Overall, despite fluctuations with intermittent peaks and troughs, the annual number of publications in the MyoG field has remained relatively stable over the past two decades, demonstrating sustained and consistent research activity.

Based on the analysis of data from 2004 to 2024 (Figure 2A), the United States, as a traditional scientific powerhouse, has maintained a leading position, publishing 36 related articles during the four-year period from 2006 to 2009. China’s research output has shown continuous growth since 2010 and reached its peak in 2022 with 12 publications, gradually emerging as a central contributor within the global research network. Japan and South Korea exhibited relatively stable research productivity, averaging 1–3 publications per year. These findings indicate that research in the MyoG field remains relatively concentrated, while emerging scientific communities are steadily expanding their presence.

### Analysis of countries/regions and institution

The global landscape of MyoG research exhibits a pronounced geographic clustering effect (Figure 2B). North America (United States: 111 publications) and East Asia (China: 97 publications) form two major centers of knowledge innovation, together accounting for 51.7% of total global output. Europe constitutes a third cluster through multi-country collaborations (Germany/France/United Kingdom/Spain: 66 publications), whereas South America (Brazil: 12 publications) and Oceania (Australia: 4 publications) occupy follower positions in the research ecosystem. This “core–periphery” distribution pattern reflects a strong association between knowledge production and national economic development as well as research investment intensity, while also highlighting the systemic challenges faced by developing countries as they attempt to catch up in frontier scientific domains.

Quality assessment based on citation metrics reveals even deeper disparities (Supplementary Material 1 Country ranking by citation counts for myogenin (MyoG) publications). The United States maintains academic leadership with a total of 4,977 citations and an average of 57.2 citations per publication, indicating substantial research depth. Notably, several medium-output countries—including the United Kingdom (53.4 citations per publication), Canada (53.1), and Italy (49.3)—maximize academic efficiency through focused research topics and extensive international collaboration. Although China, Japan, and South Korea collectively contribute 32.9% of global publications, their average citation counts (China: 13.2; Japan: 19.2; South Korea: 20.5) remain significantly lower than those of major Western countries. This “quantity–quality mismatch” underscores structural bottlenecks within East Asian research systems, particularly regarding original breakthroughs and the establishment of international academic influence. Overall, the data reflect a divergence between two innovation pathways: scale-driven expansion versus quality-oriented development.

Figure 2C presents a visualized analysis of international research collaborations, revealing a distinct “dual-core” structure dominated by China and the United States. These two countries serve as central hubs in the global collaboration network. European countries demonstrate strong regional synergy, with the United Kingdom, Germany, and France forming a cohesive regional collaboration cluster through intensive scientific interactions. Notably, developed countries such as Japan and Canada remain key nodes within the network, maintaining stable channels of knowledge exchange with major scientific powers. Meanwhile, emerging countries such as India and Brazil are gradually integrating into the international collaboration system, although their partnerships remain largely aligned with networks led by North America and Europe.

### Academic Subject analysis

#### Author analysis

By systematically examining the author collaboration network and the temporal publication patterns of prolific researchers in the MyoG field, it is possible to accurately identify the distribution of research hotspots during specific periods. Such analysis enables researchers to comprehensively understand the dynamic evolution and developmental trajectory of research themes while effectively distinguishing core contributors and their academic influence across different stages of the field’s progression.

**FIGURE 3:**
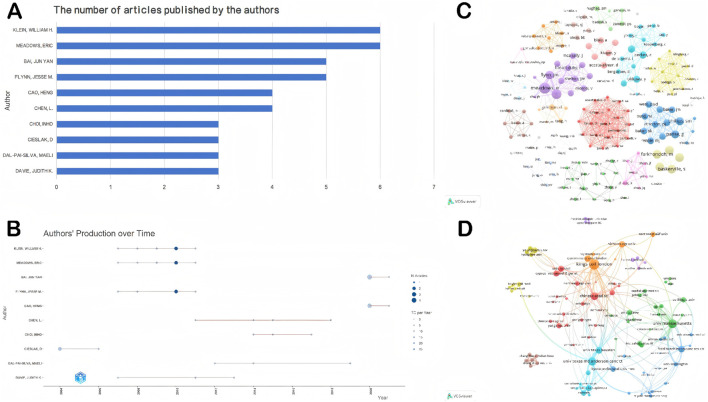
Visualizations of author productivity and collaborative networks in MyoG research. **(A)** Top ten authors ranked by publication count, highlighting the most productive contributors in the field. **(B)** Publication timeline of the top ten authors, illustrating patterns of research activity over time. **(C)** Author collaboration network, depicting partnerships and scientific interactions among researchers in the MyoG domain. **(D)** Institutional collaboration network, showing inter-institutional connections and contributions within the MyoG research landscape.

Figures 3A,B clearly present the top ten authors in terms of publication output from 2004 to 2024, along with their corresponding research timelines. Notably, Klein William H, Meadows Eric, and Fkybb Jesse M form a closely collaborating research team that has collectively published six high-quality papers in this field. As indicated by the color intensity of the timeline nodes, the team achieved a major breakthrough in 2010 with their milestone study published in Cell, which for the first time elucidated the central regulatory role of myogenin in neurogenic muscle atrophy. Their study demonstrated that denervation-induced injury upregulates myogenin expression, which in turn directly activates the transcription of the E3 ubiquitin ligase–encoding genes MuRF1 and atrogin-1, thereby driving muscle protein degradation and the atrophic process. This groundbreaking discovery not only revealed the dual biological function of myogenin—serving as a key developmental regulator during embryonic myogenesis and functioning as a molecular switch for neurogenic atrophy in adulthood—but also established a cascade signaling axis linking epigenetic regulation (HDAC4/5), transcriptional control (MyoG), and protein degradation (MuRF1/atrogin-1). This pathway provided an entirely new paradigm for understanding the pathological mechanisms underlying neuromuscular interaction [7]. The article has accumulated a total of 357 citations, underscoring its substantial impact on the field.

Figure 3C presents the overall structure of research collaborations in the MyoG field through a visualized network. In this network, node size is positively correlated with the total citation count of each researcher, providing an intuitive measure of individual academic influence. The analysis reveals a pronounced clustering pattern, indicating that scholarly activity within this field is largely organized into distinct academic groups.

#### Institutional analysis

The analysis of institutional publication output, combined with the visualization of inter-institutional collaboration networks, provides insights into the distribution of major research forces and collaborative patterns within the MyoG field. Such analyses offer valuable references for identifying core research institutions and their partnership structures.

According to the rankings in Supplementary Material 2 Top 10 Institutions by Publication Volume and Citation VolumeSichuan Agricultural University leads the field with ten publications. However, its total citation count is only 134, which is markedly lower than that of the Massachusetts Institute of Technology (MIT) and the Whitehead Institute for Biomedical Research—each of which has published only two papers but achieved a high citation count of 786. This discrepancy highlights a nonlinear relationship between academic influence and publication volume in MyoG research.

Further integration with the institutional collaboration network visualization in Figure 3D, which displays the top 100 most-cited institutions, shows that node size reflects institutional centrality within the collaborative network. Despite its high publication output, Sichuan Agricultural University occupies a peripheral position in the network. In contrast, institutions such as King’s College London, the University of Massachusetts, and the University of Texas MD Anderson Cancer Center occupy central hub positions by establishing dense collaborative networks.

The visualization also reveals several representative collaboration clusters.

King’s College London and Queen Mary University of London form a strong partnership, focusing primarily on key genes and signaling pathways involved in skeletal muscle development as well as the mechanisms underlying related pathologies such as muscle atrophy, muscle injury, and rhabdomyosarcoma. Their joint efforts also explore innovative gene therapy strategies based on gene editing and viral vectors [8, 9].

Knowledge exchange between the University of Texas MD Anderson Cancer Center and the University of Texas at Houston centers on integrating multidisciplinary expertise to investigate MyoG-mediated regulatory mechanisms during skeletal muscle development, as well as its roles in muscle metabolism and muscle disorders [10–12].

In terms of funding structures, MyoG research is predominantly supported by major public funding bodies in North America, East Asia and Europe. The top 20 funding agencies acknowledged in the 402 publications are summarised in Supplementary Material 3 Top 20 funding agencies supporting myogenin (MyoG) research. The US National Institutes of Health (NIH) and the US Department of Health and Human Services (HHS) together account for the largest share of funded papers, followed by the National Natural Science Foundation of China (NSFC), Japan’s MEXT/KAKENHI programmes, and European and national research councils such as UKRI, DFG, CIHR and others.

#### Journal analysis

By analyzing journals with high publication output and high H-index values in the MyoG field, it becomes possible to accurately identify core academic platforms and provide strategic guidance for research dissemination and scholarly collaboration.

As shown in Supplementary Material 4 Publication Volume and Related Indices in the MyoG Field Journal of Biological Chemistry (United States) leads the field with an H-index of 15 and a total of 844 citations, supported by 18 publications that underscore its dual role as a central hub for knowledge production. PLOS ONE (United Kingdom) ranks second with a stable H-index of 12. Notably, the German journal Cell and Tissue Research demonstrates a striking “low-output, high-impact” pattern: despite an H-index of only 4, it has accumulated 678 total citations, yielding an exceptional average of 169.5 citations per article—surpassing even the leading journal’s average of 46.9 citations. This phenomenon further highlights the asymmetric relationship between academic influence and publication quantity in the MyoG field, emphasizing the pivotal role of high-quality, highly targeted studies in driving advances at the disciplinary frontier.

#### Experimental models used in MyoG research

To provide an accessible overview of how MyoG has been studied experimentally, we summarised the main experimental models used in the 402 publications (Table 2). C2C12 myoblasts were by far the dominant *in vitro* system (90/402, 22.4%), whereas other cell-based models, including primary mouse myoblasts/myotubes, human skeletal myoblasts/myotubes and rhabdomyosarcoma cell lines, were used only occasionally (1.5–2.0% of records each). Among *in vivo* approaches, denervation-induced atrophy models were the most frequently employed animal models (20/402, 5.0%), followed by disease-specific settings such as Duchenne muscular dystrophy (mdx) mice and chemical injury models using cardiotoxin or BaCl_2_. Human skeletal muscle biopsies appeared in only a very small subset of studies (2/402, 0.5%), and no records in our dataset explicitly reported the use of rhabdomyosarcoma tumour specimens in the title, abstract or keyword fields.

### Content theme analysis

#### Keyword analysis

**FIGURE 4:**
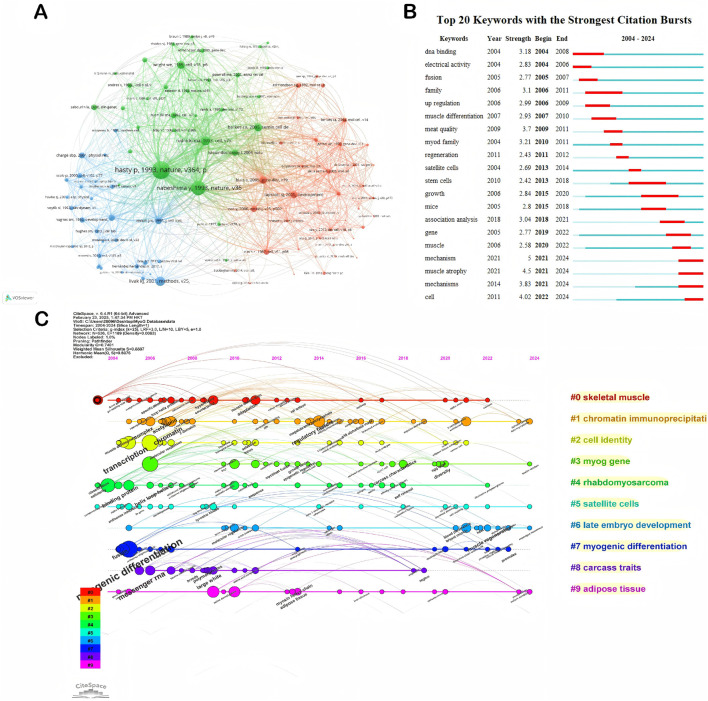
Visualizations of the publication trends and collaborative networks in MyoG research. **(A)** Co-citation analysis of references, visualizing the clustering of key research topics and their interconnections in the MyoG field. **(B)** Timeline of citation bursts for the top 20 keywords, highlighting emerging research trends and their periods of significant influence. **(C)** Timeline visualization of keywords, illustrating the evolution and prominence of research themes over time in the MyoG domain.

Keyword burst detection provides a systematic means of revealing the dynamic evolution of research hotspots within the field. Figure 4B presents the top 20 keywords with the strongest citation bursts in MyoG research from 2004 to 2024. The year of first appearance and the duration of each burst period clearly reflect the stage-specific transitions in research focus over time.

#### Theme evolution

Using the keyword co-occurrence timeline shown in Figure 4C, the ten major thematic clusters in MyoG research can be visualized intuitively. Overall, studies in this field can be categorized into three overarching directions: molecular regulatory networks, developmental and regenerative mechanisms, and disease-related research.

As a core transcription factor governing skeletal muscle differentiation and development, foundational investigations of MyoG focus on its gene-regulatory characteristics (myog gene) and its interactions within the myogenic regulatory factor network, including myod. Techniques such as chromatin immunoprecipitation (ChIP) are frequently employed to elucidate its DNA-binding patterns and epigenetic regulatory mechanisms.

At the tissue-development level, research on satellite cell activation and the temporal regulation of late embryo development has identified key nodes through which MyoG orchestrates myogenic differentiation. In translational contexts, the scope extends to pathological mechanisms, such as the aberrant regulation of MyoG-related signaling pathways in rhabdomyosarcoma, underscoring its significant clinical relevance.

### Citation and impact analysis

#### Literature citation analysis

Supplementary Material 5 Top 10 most cited MyoG-related papers (total citation counts). provides an intuitive overview of the most influential articles in the MyoG field. The 2006 publication by Rao, Prakash K and colleagues, entitled “Myogenic factors that regulate expression of muscle-specific microRNAs,” ranks first, with a total of 573 citations and an average of 28.65 citations per year. This study demonstrated that the myogenic factors myogenin (MyoG) and MyoD regulate the expression of muscle-specific microRNAs (miRNAs)—including miR-1, miR-133, and miR-206—by directly binding to the upstream regulatory regions of their genes during muscle differentiation, thereby providing important mechanistic insights into the molecular regulation of myogenesis.

#### Co-citation analysis

Reference co-citation refers to the phenomenon in which two or more publications are cited together by subsequent articles, implying that these co-cited works may share similarities or relevance in terms of research topics, methodologies, or conceptual viewpoints. Through visualized clustering analysis of co-cited references (Figure 4A), it is possible to accurately identify foundational or breakthrough studies within the MyoG field. In this network, node size represents the citation frequency of a given reference, while the thickness of the connecting lines reflects the strength of the co-citation relationship between pairs of articles. In parallel, Supplementary Material 2, provides an intuitive ranking of citation counts, further underscoring the pivotal role of these references in MyoG research.

The analysis highlights, in particular, the landmark contribution of the 1993 study by Paul Hasty and colleagues published in Nature, which investigated the global phenotype of MyoG gene knockout mice and emphasized the essential role of MyoG in skeletal muscle differentiation and formation [13]. Similarly, the Nature paper by Yoko Nabeshima and co-workers focused on alterations in muscle tissues during embryonic development in MyoG knockout mice, underscoring the critical requirement of MyoG for proper muscle formation in the embryonic stage [14]. Together, these two gene knockout studies unequivocally demonstrated the indispensable role of MyoG in skeletal muscle development, and their complementary conclusions provide important clues for understanding the regulation of muscle-specific gene expression, the pathogenesis of muscle diseases, and the interplay between muscle and skeletal development.

## Discussion

Using a bibliometric approach, this study analyzes nearly two decades of publications in the MyoG field, providing a comprehensive overview of global research collaboration, recent advances, and emerging trends. In particular, it visualizes current hotspots and developmental trajectories in MyoG research based on published records.

### Historical development

This study analyzed publications in the MyoG field from 2004 to 2024 and examined in depth the three major peaks in annual output over this 20-year period. In combination with Supplementary Material 5, it is evident that each publication peak coincided with substantial theoretical or technological advances related to MyoG. From the perspective of thematic evolution, these three peaks delineate a clear trajectory of deepening research: initial efforts focused on elucidating the physiological functions of MyoG, subsequently expanding to the dissection of disease mechanisms, and ultimately converging on injury repair. This stepwise progression has gradually driven MyoG-related research toward clinical application.

The first peak occurred in 2006 (25 publications), when researchers intensively explored the molecular mechanisms by which MyoG regulates muscle differentiation, including the pivotal role of chromatin remodeling, transcriptional regulatory networks, and epigenetic control. Ohkawa et al. conducted a series of refined molecular biology experiments that clearly demonstrated how myogenin (MyoG) cooperates with other transcription factors such as MyoD and MEF2 to regulate the expression of muscle-specific genes, thereby elucidating in depth the mechanistic role of MyoG in myogenic differentiation [15, 16]. In addition, de la Serna et al. showed that MyoD recruits the SWI/SNF chromatin-remodeling complex to the myogenin locus, promoting chromatin structural changes that create a permissive environment for MyoG expression [17]. This finding further underscored the critical importance of chromatin remodeling in muscle differentiation and clarified the interaction between chromatin-remodeling complexes (such as SWI/SNF) and muscle regulatory factors.

Building on this, Blais et al. reconstructed the transcriptional regulatory network governing muscle differentiation, revealing how muscle regulatory factors drive myogenesis through multilayered control mechanisms [18]. Collectively, these studies greatly deepened our understanding of the molecular basis of muscle differentiation and provided new potential targets and strategies for the treatment of muscle-related diseases, such as muscular dystrophies.

The second pronounced peak occurred between 2011 and 2014, with an average of 23 publications per year. As indicated by the highly cited articles summarized in Supplementary Material 1 research during this period primarily focused on breakthroughs in epigenetic regulation, deeper elucidation of molecular mechanisms, and the disease relevance and therapeutic potential of MyoG. Studies by Stojic et al. and Huang et al. provided new conceptual support for the role of epigenetic regulation in muscle development [19, 20]. In the context of disease association and therapeutic implications, Moresi et al. demonstrated that myogenin (MyoG) promotes muscle atrophy by activating E3 ubiquitin ligases, with HDAC4 and HDAC5 serving as critical regulatory nodes in this process [7]. These findings not only broadened the functional spectrum of MyoG but also offered new theoretical foundations and potential targets for the treatment of muscle disorders.

From 2020 onward, interest in MyoG research has risen once again, culminating in a new peak of 25 publications in 2022. As shown in Supplementary Material 7 Top 10 MyoG-related papers ranked by normalised citation counts, this recent surge is largely driven by growing attention to the interface between epigenetic regulation, muscle development, and disease. While researchers continue to refine our understanding of the classical role of myogenin in skeletal muscle development, increasing emphasis has been placed on its potential in cardioprotection and cardiac regeneration. In the cardiac field, our previous work demonstrated that myogenin can attenuate oxidative stress–induced apoptosis by modulating DUSP13 and p38 MAPK signaling, thereby reducing myocardial injury and promoting tissue repair [21]. Furthermore, studies centered on angiotensin II have revealed additional anti-apoptotic effects of myogenin in cardiovascular disease models, opening new avenues for therapeutic intervention [22]. Together, these emerging hotspots indicate that myogenin not only plays a pivotal role in muscle growth and development but also holds substantial promise in muscle injury repair and the treatment of cardiovascular and muscle-related diseases.

### Thematic evolution

Systematic analysis of keyword bursts (Figure 4B) intuitively illustrates how research themes in the MyoG field have progressively deepened over time.

In the early stage (2004–2011), studies primarily focused on elucidating the molecular mechanisms and fundamental functions of MyoG. Key advances included the repeated validation of DNA binding as core evidence for MyoG’s activity as a transcription factor [23]. The emergence of keywords such as MyoD family and other family-related terms reflected systematic efforts to dissect the regulatory networks and evolutionary relationships of the gene family to which MyoG belongs. During this period, investigations into molecular interactions and signaling pathways laid the theoretical foundation for understanding the mechanisms of muscle differentiation.

During this period, basic research centered on skeletal muscle entered a peak phase. Around 2004, the main focus was on MyoG-mediated regulation and activation of skeletal muscle–specific genes. High-frequency occurrence of keywords such as MyoD and activation, together with terms like acetylcholine receptor (AChR) and citrate synthase, reflected growing interest in both transcriptional control and muscle metabolism. These studies showed that MyoG serves as the sole bridge through which muscle LIM protein (MLP) participates in the regulation of the AChR gene, and plays a specialized role in the selective control of AChR expression. At the same time, the involvement of enzymes such as citrate synthase indicated that research had begun to extend into neuromuscular junction function and muscle energy metabolism. Collectively, these findings established an initial link between MyoG and skeletal muscle physiological function and challenged the earlier notion that MyoG expression is restricted to the embryonic period [24, 25].

From 2005 to 2013, the focus gradually shifted from basic transcriptional regulation to mechanisms of skeletal muscle differentiation and regeneration. The clustered emergence in 2009 of keywords such as satellite cell, hypertrophy, and beta 1 integrin directly reflected growing interest in satellite cell regulation and the mechanisms of muscle hypertrophy, and further uncovered the role of MyoG in initiating muscle regeneration [26–31]. By 2011, attention turned to the fine-tuning of the regenerative process itself. The appearance of keywords such as regeneration, methylation, and adaptation signaled that MyoG-related research had entered the epigenetic level, with the field beginning to explore how epigenetic modifications—particularly DNA methylation—regulate MyoG function and shape adaptive responses during muscle regeneration [12, 32–34].

In the intermediate phase (2013–2018), the thematic evolution of the field was characterized by a clear technological and conceptual shift toward regenerative medicine. The concentrated emergence of keywords such as satellite cells, stem cells, and regeneration indicated that the research focus had moved toward mechanisms of muscle injury repair. Notably, the high frequency of the keyword mice underscored the central role of *in vivo* animal models as a critical platform for validating the roles of MyoG in stem cell regulation and muscle regeneration.

As a key regulator of muscle-specific gene transcription, myogenin (MyoG) exerts indispensable functions across multiple stages of the muscle injury–repair process. On one hand, MyoG drives the core repair program by precisely recruiting transcription initiation complexes—such as TATA-binding protein (TBP) and RNA polymerase II—to the promoters of repair-related target genes, thereby directly activating downstream gene expression and initiating the regenerative response following muscle injury [35]. On the other hand, MyoG promotes myofiber regeneration by facilitating myocyte fusion. It does so by directly binding to and activating the myomaker gene, a critical effector of myoblast fusion whose encoded protein is essential for this process. Loss of MyoG results in a permanent defect in myocyte fusion, which in turn impedes myofiber formation and regeneration [36].

MyoG also plays a central role in regulating satellite cell function and maintaining muscle homeostasis. As illustrated in Figure 4C, satellite cells constitute one of the core themes in MyoG-related research. Current evidence indicates that MyoG exerts complex but crucial control over satellite cell behavior, not only promoting terminal stages of myogenic differentiation (such as myocyte fusion) but also helping to preserve the quiescent state of satellite cells. MyoG deletion leads to premature exit from quiescence, dysregulation of the mTORC1 signaling pathway, and aberrant interactions between satellite cells and their myofiber niche [35, 37].

In the recent phase (2019–2024), the field has increasingly shifted toward clinical translation and precision medicine. Strong citation bursts for keywords such as muscle atrophy (strength 4.5) and mechanism (strength 5.0) point to intensified efforts to dissect the pathophysiological mechanisms underlying age-related muscle degenerative diseases. Concurrently, the prominence of terms such as association analysis and gene reflects the widespread application of multi-omics technologies and gene association analyses in elucidating disease mechanisms. This stage is characterized by a clear transition from purely mechanistic studies toward research explicitly driven by clinical diagnostic and therapeutic needs.

Consistent with this late-stage shift toward clinically oriented research, analysis of disease-related research themes in the MyoG literature (Table 1) further highlighted the dominance of clinically focused topics. Among these topics, “Duchenne muscular dystrophy” alone accounted for almost half of all records (47.8%), followed by “Muscle wasting” (8.2%) and “Sarcoma research”, which includes rhabdomyosarcoma-related studies (4.0%). Together, these categories indicate that a substantial proportion of MyoG work has moved downstream from developmental biology into degenerative muscle disease, inherited dystrophies and muscle cancers. At the same time, mechanistically oriented topics such as “Epigenetic regulation”, “lncRNA”, “Hippo pathway” and “HIF in cancer” remain highly represented, suggesting that disease-oriented studies are tightly coupled to investigations of MyoG-centred regulatory pathways rather than being purely descriptive.

**TABLE 1:** Distribution of disease-related research themes in myogenin (MyoG) publications (2004–2024).

Citation Topics Micro	Records (n)	Proportion of all 402 records (%)
Duchenne muscular dystrophy	192	47.761
Muscle wasting	33	8.209
Sarcoma research	16	3.98
Epigenetic regulation	16	3.98
Microrna in cancer	8	1.99
Nicotinic receptors	7	1.741
Lncrna	6	1.493
Neural crest	4	0.995
Hippo pathway	4	0.995
Gh/igf axis	3	0.746
Bacterial gene regulation	3	0.746
Protease inhibitors	3	0.746
Heart failure management	2	0.498
Hif in cancer	2	0.498
Tgf-beta	2	0.498

Within this context, rhabdomyosarcoma (RMS) has emerged as a particularly prominent focus. RMS is a muscle-derived sarcoma that occurs predominantly in children, whose tumor cells aberrantly express myogenic regulatory factors such as MYOD1 and MYOG. Functional suppression of MyoG blocks the myogenic differentiation program and locks tumor cells in a proliferative state, constituting one of the key mechanisms driving RMS pathogenesis [38, 39]. Building on this central mechanism, multiple targeted therapeutic strategies have been developed. Leveraging the high expression of MYOG in RMS cells, researchers have engineered MYOG promoter–driven oncolytic viruses—such as Ad5/3-pMYOG (S)-mMEF2—to achieve selective oncolysis of RMS cells [40]. In parallel, therapeutic approaches that interfere with negative regulators of MYOG, such as TRPS1 and SNAI2, and modulate associated signaling pathways, including the ERK/MAPK axis, can relieve repression of MYOG function and promote the differentiation of RMS cells toward a more normal myogenic phenotype [41, 42]. Furthermore, studies on MYOG-related RNA molecules—for example, the natural antisense transcript MYOG-NAT—have begun to elucidate how RNA-based mechanisms regulate MYOG protein stability, providing a theoretical foundation for novel RNA-targeted therapeutic strategies [43].

Collectively, these advances not only substantiate MYOG and its regulatory network as promising therapeutic targets for RMS but also propel concrete gene therapy approaches—particularly oncolytic virotherapy—into the experimental stage. Future research should focus on optimizing the specificity, efficacy, and clinical safety of these strategies to develop effective MYOG-centered interventions for RMS and related malignancies [38].

The experimental landscape summarised in Table 2 further contextualises these thematic trajectories. The field remains heavily reliant on a limited set of preclinical systems, with more than one fifth of all publications using C2C12 myoblasts and a substantial fraction relying on rodent denervation models for *in vivo* validation. By contrast, human skeletal muscle biopsies and clinically proximate disease models are used only sporadically. This imbalance highlights both the strength and the limitation of current MyoG research: mechanistic insights are largely derived from tractable rodent and cell-line models, whereas translation to human pathology still depends on relatively few studies. Future work will therefore need to expand the use of human tissue, advanced genetic disease models and clinically annotated cohorts to fully exploit the translational potential outlined by our bibliometric mapping.

**TABLE 2:** Experimental models used in myogenin (MyoG) research (2004–2024).

Model category	Representative models	No. of publications (n, %)
Cell lines	C2C12 myoblasts	90 (22.4%)
	Primary mouse myoblasts/myotubes	8 (2.0%)
	Human skeletal myoblasts/myotubes	6 (1.5%)
	Rhabdomyosarcoma cell lines (e.g., RD, RH30, RH41)	8 (2.0%)
Animal models	Conventional MyoG knockout mice	2 (0.5%)
	Conditional MyoG knockout/knockdown mice	2 (0.5%)
	Duchenne muscular dystrophy (mdx) models	4 (1.0%)
	Denervation-induced atrophy models	20 (5.0%)
	Chemical injury models (cardiotoxin/BaCl_2_)	4 (1.0%)
Human samples	Human skeletal muscle biopsies	2 (0.5%)

Overall, MyoG research from 2004 to 2024 exhibits a clear pattern of staged evolution: early work concentrated on transcription factor function and gene regulatory networks; intermediate studies shifted toward mechanisms of muscle regeneration; and recent efforts have delved into the pathogenesis of muscle atrophy and driven advances in clinical translation. The regulatory axis centered on MyoG—DNA binding → MyoD family → muscle differentiation → muscle atrophy—integrates molecular interactions with disease phenotypes into a coherent dynamic continuum.

Taken together, the observed thematic evolution of MyoG research appears to mirror broader scientific and clinical drivers in muscle biology. The early dominance of transcriptional regulation and chromatin remodelling reflects the rapid expansion of molecular genetics and epigenetics in the early 2000s, when establishing MyoG as an indispensable regulator of myogenic differentiation was a central conceptual goal. The subsequent shift toward satellite cell biology and regenerative mechanisms coincides with the emergence of stem cell–based regenerative medicine and increasing interest in functional recovery after muscle injury. More recently, the prominence of keywords related to muscle atrophy, Duchenne muscular dystrophy and rhabdomyosarcoma suggests a field-level realignment toward clinically salient phenotypes, driven by population ageing, the unmet need in degenerative muscle disease and advances in genetic and cellular therapies. This trajectory—from basic regulatory circuits to regeneration and disease-focused applications—is consistent with bibliometric trajectories reported in sarcopenia, exercise and mental health research, where early mechanistic work progressively gives way to translational and precision medicine–oriented themes [44].

### Core authors

In scientific research, core teams within a field often serve as the driving force behind continuous advances at the academic frontier. Based on the visualized author collaboration network in Figure 3C, several key research clusters can be identified in the MyoG domain. Among them, Eric Meadows from The University of Texas emerges as a major central node, with seven publications and a total of 797 citations. His work primarily focuses on the functions of MyoG in muscle biology and its underlying regulatory mechanisms. Using a range of genetic approaches—including gene knockout, gene knockdown, and conditional knockout—his group has systematically dissected the roles of MyoG in muscle denervation, muscle atrophy, adult muscle stem cell function, exercise capacity and metabolism, and Duchenne muscular dystrophy models [7, 10–12, 45].

Another important research network is centered around S.J. Tapscott and C.A. Berkes at the Massachusetts Institute of Technology (MIT), whose four key publications have collectively received 702 citations. Tapscott’s work focuses on the molecular mechanisms of muscle differentiation, elucidating the sequential regulation of shared target genes by MyoD and MyoG during myogenesis. Subsequent studies from this group proposed a two-step model in which MyoD activates MyoG gene expression, thereby clarifying the dynamic changes in gene regulation that occur during muscle differentiation and providing important insights into how myogenic cells achieve differentiation through precisely orchestrated molecular programs [17, 46–48].

The joint team of S.M. Phillips and G. Parise at McMaster University in Canada also stands out, with two publications that have accumulated 519 citations, making them another major source of high-impact knowledge production in North America. Although these core teams are geographically dispersed, their highly influential contributions collectively form an implicit academic coordinate system that structures the intellectual landscape of MyoG research.

### Future perspectives

Our findings also need to be interpreted in the context of the wider bibliometric landscape of muscle and ageing research. Recent mapping studies on sarcopenia, exercise and frailty, and mental health–related digital research have consistently reported a similar macro-structure, with a small number of high-income countries and specialised centres dominating knowledge production and collaboration networks, and emerging regions contributing growing but comparatively lower-impact outputs. Within this pattern, MyoG research appears to occupy a more upstream, mechanism-focused niche that feeds into downstream themes such as sarcopenia, muscle wasting and functional decline. The pronounced “quantity–quality imbalance” we observed in East Asian outputs therefore echoes imbalances reported in sarcopenia and exercise bibliometrics, and reinforces the need for strategic investment in cross-centre collaboration, longitudinal cohorts and multi-omics platforms if mechanistic insights around MyoG are to translate into clinically meaningful gains.

Drawing on the bibliometric analysis of MyoG-related literature from 2004 to 2024, which reveals clear patterns of thematic evolution, core bottlenecks, and potential breakthrough points, and considering the practical demands of muscle development regulation, regenerative medicine, and clinical translation, future research on MyoG should advance in a coordinated manner along three interconnected axes—mechanistic deepening, technological innovation, and clinical implementation. Such an integrated strategy will be essential to fully realize the central value of MyoG in both fundamental research and disease intervention.

Currently, MyoG research has shifted from basic functional exploration toward a stronger focus on pathological mechanisms, yet the integrity and specificity of disease-related regulatory networks remain incomplete. Future work should prioritize major conditions such as age-related muscle atrophy, Duchenne muscular dystrophy, and rhabdomyosarcoma (RMS), with particular emphasis on the following directions.

First, epigenomic approaches such as chromatin immunoprecipitation sequencing (ChIP-seq) and assay for transposase-accessible chromatin using sequencing (ATAC-seq) should be leveraged to dissect the dynamic interactions between MyoG, histone modifications, and DNA methylation. This will help identify “switch-like” regulatory nodes through which MyoG controls disease progression, with a special focus on clarifying the cascade regulation between MyoG and the HDAC4/5–E3 ubiquitin ligase axis in denervation-induced injury, thereby providing novel targets for the selective inhibition of muscle atrophy [49].

Second, the mechanisms underlying functional suppression of MyoG in RMS require deeper investigation. Future studies should concentrate on the cooperative effects of inhibitory factors such as TRPS1 and SNAI2 with the ERK/MAPK signaling pathway, and delineate how the natural antisense transcript of MyoG (MYOG-NAT) regulates MyoG protein stability. Deciphering these processes will be key to resolving the “differentiation block” characteristic of tumor cells and will provide a mechanistic basis for precisely relieving MyoG inhibition in RMS [41–43].

Existing MyoG research is still constrained by limitations in dissecting cellular heterogeneity and in modeling disease-relevant phenotypes, and these gaps will require technological innovation to overcome. Moving forward, the integration of single-cell, spatial, and genome-editing technologies should be actively promoted. On the one hand, combining single-cell RNA sequencing (scRNA-seq) with spatial transcriptomics will enable construction of spatiotemporal expression atlases of MyoG under distinct physiological and pathological conditions—such as quiescent versus activated satellite cells and different stages of myofiber injury and repair—thereby resolving cell subtype–specific regulatory networks and helping to reconcile the apparent paradox between maintaining satellite cell homeostasis and ensuring efficient myocyte fusion [37]. On the other hand, CRISPR-Cas9–based gene editing can be used to generate conditional MyoG knockout or mutation animal models that recapitulate clinically relevant genetic alterations (e.g., MyoG promoter mutations in RMS), providing a more precise alternative to traditional germline knockout models and enhancing the fidelity of drug screening and mechanistic validation [50]. In parallel, the application of artificial intelligence to MyoG regulatory network prediction—through integrative modeling of multi-omics datasets (genomics, transcriptomics, metabolomics)—may accelerate the discovery of previously unrecognized MyoG-interacting molecules and signaling pathways.

The complexity of MyoG-centered regulatory networks extends beyond the protein level; the potential contributions of noncoding RNAs and metabolic pathways remain insufficiently explored. Future work should therefore systematically advance along two major lines. First, with respect to noncoding RNAs—including microRNAs (miRNAs), long noncoding RNAs (lncRNAs), and circular RNAs (circRNAs)—RNA sequencing (RNA-seq) combined with RNA pull-down assays should be employed to identify and validate key molecules that regulate MyoG expression, and to clarify their roles in muscle regeneration (e.g., satellite cell activation) and disease (e.g., muscle atrophy). Particular emphasis should be placed on deepening our understanding of the interactions between miR-1/133 and MyoG, with the goal of developing RNA interference–or mimetic-based tools for precise modulation of MyoG activity [41, 43]. Second, the interplay between metabolic reprogramming and MyoG function warrants in-depth investigation. Future studies should focus on how glycolysis and fatty acid oxidation pathways influence MyoG-mediated myocyte differentiation and fusion, and determine whether metabolic signaling cascades such as AMP-activated protein kinase (AMPK) and mechanistic target of rapamycin complex 1 (mTORC1) contribute to muscle homeostasis by modulating MyoG activity. Such insights could provide a new conceptual framework for improving muscle function through metabolic interventions, including exercise and nutritional modulation [51].

The translation of advances in the MyoG field into clinical applications remains at an early stage, and future efforts must focus on overcoming key bottlenecks along the translational continuum of technology optimization–efficacy validation–safety evaluation. In terms of targeted therapy, existing oncolytic viral vectors—such as Ad5/3-pMYOG (S)-mMEF2—require further refinement to enhance tumor tropism and infection efficiency while minimizing toxicity to normal myocytes. In parallel, gene therapy vectors driven by the MyoG promoter should be developed to achieve selective expression of therapeutic genes in diseased muscle cells [42].

For diagnosis and prognostic assessment, large-scale clinical datasets are needed to validate the feasibility of MyoG mRNA and protein expression levels as diagnostic biomarkers and prognostic indicators for muscle diseases such as rhabdomyosarcoma (RMS) and muscle atrophy. By integrating these molecular markers with imaging modalities, including magnetic resonance imaging (MRI) and ultrasound, a combined “molecular biomarker–imaging phenotype” evaluation system could be established to enable early disease screening and dynamic monitoring of therapeutic responses.

The complexity of MyoG research and its extensive connections to multiple biological systems make interdisciplinary collaboration and international cooperation indispensable. Future work should further integrate developmental biology, immunology, metabolomics, and clinical medicine to expand the conceptual and translational boundaries of MyoG research. For example, it will be important to elucidate the role of MyoG in cardioprotection—such as its ability to suppress cardiomyocyte apoptosis via regulation of the DUSP13–p38 MAPK pathway—and in neuromuscular interactions, including neural regulation of muscle regeneration following nerve injury, thereby uncovering its potential relevance in cardiovascular and neurodegenerative diseases. In parallel, a globally accessible MyoG data-sharing platform should be established to aggregate clinical samples, experimental datasets, and research outputs from different countries and regions. Such an infrastructure would help to resolve the “quantity–quality mismatch” observed in some emerging research communities, promote more rational allocation and efficient utilization of global MyoG research resources, and accelerate overall progress in the field.

In summary, future MyoG research should be explicitly guided by the goal of addressing real-world clinical problems, driven by technological innovation, and sustained by robust interdisciplinary collaboration. By advancing mechanistic understanding and translational application in tandem, MyoG research can progressively move from basic science toward clinical implementation, providing essential support for precision therapy of muscle diseases and for the continued development of regenerative medicine.

From a translational perspective, the bibliometric patterns identified here provide a practical roadmap rather than a purely descriptive overview. The concentration of highly cited work around MyoG-dependent atrophy pathways and rhabdomyosarcoma biology suggests that future experimental studies should prioritise dissecting MyoG-centred regulatory axes that are directly druggable (e.g., HDAC–E3 ligase cascades, ERK/MAPK signalling, MYOG-NAT–mediated RNA regulation) and testing these in disease-relevant *in vivo* models. At the clinical and multidisciplinary level, our collaboration and hotspot maps highlight at least three actionable priorities: (i) building multi-centre networks that link basic myogenesis laboratories with neuromuscular, oncology and geriatric units; (ii) embedding MyoG-related biomarkers into longitudinal cohorts and interventional trials for muscle wasting and paediatric muscle tumours; and (iii) fostering cross-talk with digital and imaging sciences to integrate molecular readouts with functional and imaging phenotypes. Explicitly aligning future experimental and clinical agendas with these mapped hotspots and gaps may accelerate the translation of MyoG biology into tangible benefits for patients with muscle disease.

### Strengths and limitations

Bibliometric analysis provides a valuable window for researchers to gain an in-depth understanding of knowledge dynamics within a specific field and has become an important tool for exploring the intrinsic structure of scientific knowledge. However, this methodological approach also has several limitations. First, the literature search in this study was initially restricted to the Web of Science Core Collection and to publications in English. Such constraints may introduce selection bias; for example, the exclusion of Chinese-language publications could lead to an underestimation of contributions from Chinese researchers. Second, this work relied on multiple software tools, including CiteSpace, bibliometrics-related packages in R, and VOSviewer. Although these tools can generate broadly consistent results, some key information may still be inadvertently overlooked due to differences in algorithms or parameter settings.

In addition, bibliometric analysis itself carries inherent limitations. (i) It primarily focuses on the retrieval, screening, and organization of publications to provide a macroscopic overview of the literature, and therefore may lack sufficient depth for nuanced interpretation of scientific content. (ii) Citation-based indicators may be influenced by factors such as self-citation, language bias, and discipline-specific citation practices, which can compromise the reliability and validity of the findings. (iii) Journal-level metrics, such as the journal impact factor, may not accurately reflect the true influence of individual researchers or specific articles when used inappropriately at the micro level and can, in some cases, be misleading.

Despite these limitations, bibliometric analysis still enables a clear visualization of the complex interactions among authors, institutions, and countries, and helps to identify emerging trends and future directions in the MyoG field.

## Conclusion

This bibliometric analysis delineates the evolutionary trajectory of MyoG research from 2004 to 2024. The global scientific landscape in this field exhibits a multipolar collaborative pattern: traditional research powerhouses continue to dominate the innovative frontier, whereas emerging countries are rapidly increasing their output but still lag behind in academic influence, reflecting structural differences between basic research–driven and technology application–oriented systems. Research hotspots have progressively shifted from molecular mechanisms—such as DNA binding and interactions with MyoD—toward regenerative medicine, particularly satellite cell regulation, and more recently toward clinical translation, including the elucidation of muscle atrophy mechanisms. Landmark studies have continued to shape the conceptual foundation of the field, while recent breakthroughs have expanded the pathological and translational relevance of MyoG.

Future work should focus on deepening the understanding of molecular mechanisms underlying muscle degenerative diseases, integrating emerging technologies such as single-cell sequencing and spatial transcriptomics, and clarifying the interplay between MyoG, noncoding RNAs, and metabolic reprogramming, with the ultimate goal of advancing MyoG-targeted therapeutic strategies into clinical practice. Although this study is constrained by potential data source bias and methodological limitations—such as reliance on a single primary database and specific software tools—it nonetheless provides a panoramic overview of the MyoG research landscape. Moving forward, the integration of multiple databases, development of more refined evaluation frameworks, and reinforcement of interdisciplinary and international collaboration will be essential to optimize research pathways and unlock the full potential of MyoG in muscle development regulation, disease intervention, and regenerative medicine.

## Data Availability

The bibliometric data used in this study were retrieved from the Web of Science Core Collection (https://www.webofscience.com) on 14 February 2025. The raw records are subject to licence restrictions and cannot be shared directly by the authors, but can be obtained by any qualified researcher with institutional access to Web of Science using the search strategy described in the Methods section. Processed data (e.g., aggregated country-, institution- and keyword-level matrices) and analysis scripts are available from the corresponding author on reasonable request.

## Footnotes

1
https://www.webofscience.com

## References

[1] Davis RL Weintraub H Lassar AB . Expression of a single transfected cDNA converts fibroblasts to myoblasts. Cell (1987) 51(6):987–1000. 10.1016/0092-8674(87)90585-x 3690668

[2] Wright WE Sassoon DA Lin VK . Myogenin, a factor regulating myogenesis, has a domain homologous to MyoD. Cell (1989) 56(4):607–17. 10.1016/0092-8674(89)90583-7 2537150

[3] Sassoon D Lyons G Wright WE Lin V Lassar A Weintraub H Expression of two myogenic regulatory factors myogenin and MyoD1 during mouse embryogenesis. Nature (1989) 341(6240):303–7. 10.1038/341303a0 2552320

[4] Rantanen J Hurme T Lukka R Heino J Kalimo H . Satellite cell proliferation and the expression of myogenin and desmin in regenerating skeletal muscle: evidence for two different populations of satellite cells. Lab Investigation; a Journal Technical Methods Pathology (1995) 72(3):341–7. 7898053

[5] Azizan A . Exercise and frailty in later life: a systematic review and bibliometric analysis of research themes and scientific collaborations. Int J Popul Stud (2024) 11(1):1. 10.36922/ijps.3282

[6] Azizan A . Mapping the muscle mass: a birds-eye view of sarcopenia research through bibliometric network analysis. Int J Disabilities Sports and Health Sci (2024) 7(1):134–43. 10.33438/ijdshs.1362539

[7] Moresi V Williams AH Meadows E Flynn JM Potthoff MJ McAnally J Myogenin and class II HDACs control neurogenic muscle atrophy by inducing E3 ubiquitin ligases. Cell (2010) 143(1):35–45. 10.1016/j.cell.2010.09.004 20887891 PMC2982779

[8] Calhabeu F Hayashi S Morgan JE Relaix F Zammit PS . Alveolar rhabdomyosarcoma-associated proteins PAX3/FOXO1A and PAX7/FOXO1A suppress the transcriptional activity of MyoD-target genes in muscle stem cells. Oncogene (2013) 32(5):651–62. 10.1038/onc.2012.73 22710712

[9] Hinits Y Osborn DPS Hughes SM . Differential requirements for myogenic regulatory factors distinguish medial and lateral somitic, cranial and fin muscle fibre populations. Development (2009) 136(3):403–14. 10.1242/dev.028019 19141670 PMC2687589

[10] Meadows E Cho JH Flynn JM Klein WH . Myogenin regulates a distinct genetic program in adult muscle stem cells. Developmental Biol (2008) 322(2):406–14. 10.1016/j.ydbio.2008.07.024 18721801

[11] Flynn JM Meadows E Fiorotto M Klein WH . Myogenin regulates exercise capacity and skeletal muscle metabolism in the adult mouse. PLoS One (2010) 5(10):e13535. 10.1371/journal.pone.0013535 21042574 PMC2962629

[12] Meadows E Flynn JM Klein WH . Myogenin regulates exercise capacity but is dispensable for skeletal muscle regeneration in adult mdx mice. PLoS One (2011) 6(1):e16184. 10.1371/journal.pone.0016184 21264243 PMC3021523

[13] Hasty P Bradley A Morris JH Edmondson DG Venuti JM Olson EN Muscle deficiency and neonatal death in mice with a targeted mutation in the myogenin gene. Nature (1993) 364(6437):501–6. 10.1038/364501a0 8393145

[14] Nabeshima Y Hanaoka K Hayasaka M Esumi E Li S Nonaka I Myogenin gene disruption results in perinatal lethality because of severe muscle defect. Nature (1993) 364(6437):532–5. 10.1038/364532a0 8393146

[15] Ohkawa Y Marfella CGA Imbalzano AN . Skeletal muscle specification by myogenin and Mef2D via the SWI/SNF ATPase Brg1. Embo J (2006) 25(3):490–501. 10.1038/sj.emboj.7600943 16424906 PMC1383528

[16] Rao PK Kumar RM Farkhondeh M Baskerville S Lodish HF . Myogenic factors that regulate expression of muscle-specific. microRNAs. Proc Natl Acad Sci U S A. (2006) 103(23):8721–6. 10.1073/pnas.0602831103 16731620 PMC1482645

[17] de la Serna IL Ohkawa Y Berkes CA Bergstrom DA Dacwag CS Tapscott SJ MyoD targets chromatin remodeling complexes to the myogenin locus prior to forming a stable DNA-bound complex. Mol Cell Biol (2005) 25(10):3997–4009. 10.1128/mcb.25.10.3997-4009.2005 15870273 PMC1087700

[18] Blais A Tsikitis M Acosta-Alvear D Sharan R Kluger Y Dynlacht BD . An initial blueprint for myogenic differentiation. Genes Dev (2005) 19(5):553–69. 10.1101/gad.1281105 15706034 PMC551576

[19] Stojic L Jasencakova Z Prezioso C Stützer A Bodega B Pasini D Chromatin regulated interchange between polycomb repressive complex 2 (PRC2)-Ezh2 and PRC2-Ezh1 complexes controls myogenin activation in skeletal muscle cells. Epigenetics and Chromatin (2011) 4:16. 10.1186/1756-8935-4-16 21892963 PMC3180244

[20] Huang MB Xu H Xie SJ Zhou H Qu LH . Insulin-like growth Factor-1 receptor is regulated by microRNA-133 during skeletal myogenesis. PLoS One (2011) 6(12):e29173. 10.1371/journal.pone.0029173 22195016 PMC3240640

[21] Luo J Gao Q Qiu HL Zhang S Zou WW Wang P Myogenin regulates DUSP13 to inhibit apoptosis induced by reactive oxygen species. Front Bioscience-Landmark (2024) 29(2):49. 10.31083/j.fbl2902049 38420814

[22] Gao Q Wang P Qiu HL Qiu B Yi WJ Tu WC Myogenin suppresses apoptosis induced by angiotensin II in human induced pluripotent stem cell-derived cardiomyocytes. Biochem Biophysical Res Commun (2021) 552:84–90. 10.1016/j.bbrc.2021.03.031 33743352

[23] Zhang L Wang C . Identification of a new class of PAX3-FKHR target promoters: a role of the Pax3 paired box DNA binding domain. Oncogene (2007) 26(11):1595–605. 10.1038/sj.onc.1209958 16964289 PMC2238811

[24] Lu PY Taylor M Jia HT Ni JH . Muscle LIM protein promotes expression of the acetylcholine receptor γ-subunit gene cooperatively with the myogenin-E12 complex. Cell Mol Life Sci (2004) 61(18):2386–92. 10.1007/s00018-004-4213-x 15378207 PMC11138884

[25] Fraterman S Khurana TS Rubinstein NA . Identification of acetylcholine receptor subunits differentially expressed in singly and multiply innervated fibers of extraocular muscles. Invest Ophthalmol and Vis Sci (2006) 47(9):3828–34. 10.1167/iovs.06-0073 16936094

[26] Chen DH Chen SC Wang W Liu F Zhang CS Zheng HL . Modulation of satellite cells in rat facial muscle following denervation and delayed reinnervation. Acta Oto-Laryngologica (2010) 130(12):1411–20. 10.3109/00016489.2010.496464 20632902

[27] Yajima H Motohashi N Ono Y Sato S Ikeda K Masuda S Six family genes control the proliferation and differentiation of muscle satellite cells. Exp Cell Res (2010) 316(17):2932–44. 10.1016/j.yexcr.2010.08.001 20696153

[28] Lindström M Pedrosa-Domellöf F Thornell LE . Satellite cell heterogeneity with respect to expression of MyoD, myogenin, Dlk1 and c-Met in human skeletal muscle: application to a cohort of power lifters and sedentary men. Histochem Cell Biol (2010) 134(4):371–85. 10.1007/s00418-010-0743-5 20878332 PMC2954291

[29] Riuzzi F Sorci G Sagheddu R Donato R . HMGB1-RAGE regulates muscle satellite cell homeostasis through p38-MAPK- and myogenin-dependent repression of Pax7 transcription. J Cell Sci (2012) 125(6):1440–54. 10.1242/jcs.092163 22328527

[30] McFarland DC Pesall JE Coy CS Velleman SG . Effects of 17β-estradiol on Turkey myogenic satellite cell proliferation, differentiation, and expression of glypican-1, MyoD and myogenin. Comp Biochem Physiol A-Molecular and Integr Physiol (2013) 164(4):565–71. 10.1016/j.cbpa.2013.01.001 23319163

[31] Chisada S Okamoto H Taniguchi Y Kimori Y Toyoda A Sakaki Y Myostatin-deficient medaka exhibit a double-muscling phenotype with hyperplasia and hypertrophy, which occur sequentially during post-hatch development. Developmental Biol (2011) 359(1):82–94. 10.1016/j.ydbio.2011.08.027 21925159

[32] Le Grand F Grifone R Mourikis P Houbron C Gigaud C Pujol J Six1 regulates stem cell repair potential and self-renewal during skeletal muscle regeneration. J Cell Biol (2012) 198(5):815–32. 10.1083/jcb.201201050 22945933 PMC3432771

[33] Steffens AA Hong GM Bain LJ . Sodium arsenite delays the differentiation of C2C12 mouse myoblast cells and alters methylation patterns on the transcription factor myogenin. Toxicol Appl Pharmacol (2011) 250(2):154–61. 10.1016/j.taap.2010.10.006 20965206 PMC3014457

[34] Campos C Valente LMP Conceiçao LEC Engrola S Fernandes JMO . Temperature affects methylation of the myogenin putative promoter, its expression and muscle cellularity in Senegalese sole larvae. Epigenetics (2013) 8(4):389–97. 10.4161/epi.24178 23538611 PMC3674048

[35] Adhikari A Kim W Davie J . Myogenin is required for assembly of the transcription machinery on muscle genes during skeletal muscle differentiation. PLoS One (2021) 16(1):e0245618. 10.1371/journal.pone.0245618 33465133 PMC7815108

[36] Ganassi M Badodi S Quiroga HPO Zammit PS Hinits Y Hughes SM . Myogenin promotes myocyte fusion to balance fibre number and size. Nat Commun (2018) 9:1–17. 10.1038/s41467-018-06583-6 30315160 PMC6185967

[37] Ganassi M Badodi S Wanders K Zammit PS Hughes SM . Myogenin is an essential regulator of adult myofibre growth and muscle stem cell homeostasis. Elife (2020) 9:9doi. 10.7554/eLife.60445 33001028 PMC7599067

[38] Hsu JY Danis EP Nance S O'Brien JH Gustafson AL Wessells VM SIX1 reprograms myogenic transcription factors to maintain the rhabdomyosarcoma undifferentiated state. Cell Rep (2022) 38(5):110323. 10.1016/j.celrep.2022.110323 35108532 PMC8917510

[39] Pruller J Hofer I Ganassi M Heher P Ma MT Zammit PS . A modified human Myogenin promoter that is highly active in alveolar rhabdomyosarcoma. Cancer Gene Ther (2021) 28(5):427–41. 10.1038/s41417-020-00225-0 32973362 PMC8119243

[40] Yoshida H Sato-Dahlman M Hajeri P Jacobsen K Koodie L Yanagiba C Mutant myogenin promoter-controlled oncolytic adenovirus selectively kills PAX3-FOXO1-positive rhabdomyosarcoma cells. Translational Oncol (2021) 14(2):100997. 10.1016/j.tranon.2020.100997 33338875 PMC7749408

[41] Huettner SS Henze H Elster D Koch P Anderer U von Eyss B A dysfunctional miR-1-TRPS1-MYOG axis drives ERMS by suppressing terminal myogenic differentiation. Mol Ther (2023) 31(9):2612–32. 10.1016/j.ymthe.2023.07.003 37452493 PMC10492030

[42] Yohe ME Gryder BE Shern JF Song YK Chou HC Sindiri S MEK inhibition induces MYOG and remodels super-enhancers in RAS-driven rhabdomyosarcoma. Sci Translational Med (2018) 10 (448):1–14. 10.1126/scitranslmed.aan4470 29973406 PMC8054766

[43] Yin YQ Chen GH Lin ZT Zhang DL Lin WJ Luo W . Natural antisense transcript of MYOG regulates development and regeneration in skeletal muscle by shielding the binding sites of MicroRNAs of MYOG mRNA 3'UTR. Biochem Biophysical Res Commun (2023) 662:93–103. 10.1016/j.bbrc.2023.04.050 37104884

[44] Azizan A , Centre of Physiotherapy FoHSUTMPASM, Clinical, Rehabilitation Exercise Research Group FoHSUTMPASM. Mapping the knowledge structure of online learning research in health sciences education: a bibliometric network analysis. Education Med J (2025) 17(3):1–15. 10.21315/eimj2025.17.3.1

[45] Tang HB Macpherson P Marvin M Meadows E Klein WH Yang XJ A histone deacetylase 4/Myogenin positive feedback loop coordinates denervation-dependent gene induction and suppression. Mol Biol Cell (2009) 20(4):1120–31. 10.1091/mbc.E08-07-0759 19109424 PMC2642751

[46] Cao Y Kumar RM Penn BH Berkes CA Kooperberg C Boyer LA Global and gene-specific analyses show distinct roles for Myod and Myog at a common set of promoters. Embo J (2006) 25(3):502–11. 10.1038/sj.emboj.7600958 16437161 PMC1383539

[47] Yang ZJP Broz DK Noderer WL Ferreira JP Overton KW Spencer SL p53 suppresses muscle differentiation at the myogenin step in response to genotoxic stress. Cell Death Differ (2015) 22(4):560–73. 10.1038/cdd.2014.189 25501595 PMC4356341

[48] Olguin HC Yang ZH Tapscott SJ Olwin BB . Reciprocal inhibition between Pax7 and muscle regulatory factors modulates myogenic cell fate determination. J Cell Biol (2007) 177(5):769–79. 10.1083/jcb.200608122 17548510 PMC2064278

[49] Ma WJ Cai Y Shen YT Chen X Zhang LL Ji YA HDAC4 knockdown alleviates denervation-induced muscle atrophy by inhibiting myogenin-dependent atrogene activation. Front Cell Neurosci (2021) 15:663384. 10.3389/fncel.2021.663384 34276308 PMC8278478

[50] Long KR Li XK Su D Zeng S Li HK Zhang Y Exploring high-resolution chromatin interaction changes and functional enhancers of myogenic marker genes during myogenic differentiation. J Biol Chem (2022) 298(8):102149. 10.1016/j.jbc.2022.102149 35787372 PMC9352921

[51] Prince LM Rand MD . Methylmercury exposure causes a persistent inhibition of myogenin expression and C2C12 myoblast differentiation. Toxicology (2018) 393:113–22. 10.1016/j.tox.2017.11.002 29104120 PMC5757876

