# Supplementary material for: Global MyoG research 2004–2024: a bibliometric analysis of trends and translational implications
Source: Exp Biol Med (Maywood). 2026 Mar 5;251:10929. doi: 10.3389/ebm.2026.10929 (PMC12999542; doi:10.3389/ebm.2026.10929)
Supplement: Supplementary file 1 [file Table1.docx]

**Supplementary File 1.** Country ranking by citation counts for myogenin (MyoG) publications (2004–2024).

| **Rank** | **Country** | **Articles** | **TC** | **Average Article Citations** |
| --- | --- | --- | --- | --- |
| 1 | USA | 111 | 4977 | 57.20 |
| 2 | CHINA | 97 | 1302 | 13.20 |
| 3 | CANADA | 20 | 796 | 53.10 |
| 4 | JAPAN | 44 | 789 | 19.20 |
| 5 | ITALY | 16 | 739 | 49.30 |
| 6 | UNITED KINGDOM | 20 | 587 | 53.40 |
| 7 | SOUTH KOREA | 26 | 534 | 20.50 |
| 8 | FRANCE | 21 | 516 | 39.70 |
| 9 | SPAIN | 12 | 213 | 23.70 |
| 10 | BRAZIL | 12 | 193 | 17.50 |
