# Supplementary material for: Global MyoG research 2004–2024: a bibliometric analysis of trends and translational implications
Source: Exp Biol Med (Maywood). 2026 Mar 5;251:10929. doi: 10.3389/ebm.2026.10929 (PMC12999542; doi:10.3389/ebm.2026.10929)
Supplement: Supplementary file 2 [file Table7.docx]

**Supplementary File 7.** Top 10 MyoG-related papers ranked by normalised citation counts.

| **Rank** | **Title** | **DOI** | **year** | **Local Citations** | **Total Citations** |
| --- | --- | --- | --- | --- | --- |
| 1 | Muscle deficiency and neonatal death in mice with a targeted mutation in the myogenin gene | 10.1038/364501A0 | 1993 | 111 | 1,346 |
| 2 | Myogenin gene disruption results in perinatal lethality because of severe muscle defect | 10.1038/364532A0 | 1993 | 85 | 924 |
| 3 | Analysis of relative gene expression data using real-time quantitative PCR and the 2(-Delta Delta C(T)) Method | 10.1006/METH.2001.1262 | 2001 | 49 | 162,222 |
| 4 | MyoD or Myf-5 is required for the formation of skeletal muscle | 10.1016/0092-8674(93)90621-V | 1993 | 47 | 1,692 |
| 5 | MyoD and the transcriptional control of myogenesis | 10.1016/J.SEMCDB.2005.07.006 | 2005 | 41 | 806 |
| 6 | Myogenin, a factor regulating myogenesis, has a domain homologous to MyoD | 10.1016/0092-8674(89)90583-7 | 1989 | 40 | 1,337 |
| 7 | An initial blueprint for myogenic differentiation | 10.1101/GAD.1281105 | 2005 | 36 | 441 |
| 8 | The circuitry of a master switch: Myod and the regulation of skeletal muscle gene transcription | 10.1242/DEV.01874 | 2005 | 34 | 721 |
| 9 | MYOGENIN IS REQUIRED, FOR LATE BUT NOT EARLY ASPECTS OF MYOGENESIS DURING MOUSE DEVELOPMENT | 10.1083/JCB.128.4.563 | 1995 | 32 | 291 |
| 10 | Mrf4 determines skeletal muscle identity in Myf5:Myod double-mutant mice | 10.1038/NATURE02876 | 2004 | 30 | 644 |
