# Supplementary material for: Global MyoG research 2004–2024: a bibliometric analysis of trends and translational implications
Source: Exp Biol Med (Maywood). 2026 Mar 5;251:10929. doi: 10.3389/ebm.2026.10929 (PMC12999542; doi:10.3389/ebm.2026.10929)
Supplement: Supplementary file 3 [file Table2.docx]

**Supplementary File 2.** Top 10 institutions ranked by publication volume and total citations in the MyoG field.

| **Articles Rank** | **Organization** | **Articles** | **Total Cited** | **Cited Rank** | **Organization** | **Articles** | **Total Cited** |
| --- | --- | --- | --- | --- | --- | --- | --- |
| 1 | sichuan agr univ | 10 | 134 | 1 | mit | 2 | 786 |
| 2 | polish acad sci | 7 | 140 | 2 | whitehead inst biomed res | 2 | 786 |
| 3 | kings coll london | 6 | 409 | 3 | univ texas md anderson canc ctr | 5 | 619 |
| 4 | kyoto prefectural univ med | 6 | 171 | 4 | mcmaster univ | 3 | 557 |
| 5 | northeast agr univ | 6 | 100 | 5 | fred hutchinson canc res ctr | 4 | 521 |
| 6 | northwest a&f univ | 6 | 65 | 6 | univ massachusetts | 4 | 436 |
| 7 | univ michigan | 6 | 325 | 7 | kings coll london | 6 | 409 |
| 8 | yangzhou univ | 6 | 56 | 8 | univ texas sw med ctr dallas | 2 | 401 |
| 9 | chinese acad sci | 5 | 69 | 9 | univ nottingham | 1 | 386 |
| 10 | henan univ sci & technol | 5 | 5 | 10 | nyu | 2 | 379 |
