# Supplementary material for: Global MyoG research 2004–2024: a bibliometric analysis of trends and translational implications
Source: Exp Biol Med (Maywood). 2026 Mar 5;251:10929. doi: 10.3389/ebm.2026.10929 (PMC12999542; doi:10.3389/ebm.2026.10929)
Supplement: Supplementary file 4 [file Table5.docx]

**Supplementary File 5.** Top 10 most cited MyoG-related papers (total citation counts).

| **RANK** | **Title** | **Doi** | **Year** | **Total Citations** | **TC per Year** |
| --- | --- | --- | --- | --- | --- |
| 1 | Myogenic factors that regulate expression of muscle-specific microRNAs | 10.1073/pnas.0602831103 | 2006 | 573 | 28.65 |
| 2 | Low-load high volume resistance exercise stimulates muscle protein synthesis more than high-load low volume resistance exercise in young men | 10.1371/journal.pone.0012033 | 2010 | 386 | 24.13 |
| 3 | An initial blueprint for myogenic differentiation | 10.1101/gad.1281105 | 2005 | 366 | 17.43 |
| 4 | Myogenin and class II HDACs control neurogenic muscle atrophy by inducing E3 ubiquitin ligases | 10.1016/j.cell.2010.09.004 | 2010 | 357 | 22.31 |
| 5 | Reciprocal inhibition between Pax7 and muscle regulatory factors modulates myogenic cell fate determination | 10.1083/jcb.200608122 | 2007 | 231 | 12.16 |
| 6 | MyoD targets chromatin remodeling complexes to the myogenin locus prior to forming a stable DNA-bound complex | 10.1128/MCB.25.10.3997-4009.2005 | 2005 | 225 | 10.71 |
| 7 | Global and gene-specific analyses show distinct roles for Myod and Myog at a common set of promoters | 10.1038/sj.emboj.7600958 | 2006 | 213 | 10.65 |
| 8 | Myostatin inhibits myogenesis and promotes adipogenesis in C3H 10T(1/2) mesenchymal multipotent cells | 10.1210/en.2005-0362 | 2005 | 171 | 8.14 |
| 9 | Microrna-221 and microrna-222 modulate differentiation and maturation of skeletal muscle cells | 10.1371/journal.pone.0007607 | 2009 | 170 | 10.00 |
| 10 | The development of genipin-crosslinked poly(caprolactone) (PCL)/gelatin nanofibers for tissue engineering applications | 10.1002/mabi.200900168 | 2010 | 147 | 9.19 |
