# Supplementary material for: Global MyoG research 2004–2024: a bibliometric analysis of trends and translational implications
Source: Exp Biol Med (Maywood). 2026 Mar 5;251:10929. doi: 10.3389/ebm.2026.10929 (PMC12999542; doi:10.3389/ebm.2026.10929)
Supplement: Supplementary file 5 [file Table3.docx]

**Supplementary Table S3** Top 20 funding agencies supporting myogenin (MyoG) research.

| **Rank** | **Funding agency** | **Number of funded publications** |
| --- | --- | --- |
| 1 | NATIONAL INSTITUTES OF HEALTH NIH USA | 71 |
| 2 | UNITED STATES DEPARTMENT OF HEALTH HUMAN SERVICES | 71 |
| 3 | NATIONAL NATURAL SCIENCE FOUNDATION OF CHINA NSFC | 42 |
| 4 | MINISTRY OF EDUCATION CULTURE SPORTS SCIENCE AND TECHNOLOGY JAPAN MEXT | 21 |
| 5 | NIH NATIONAL CANCER INSTITUTE NCI | 19 |
| 6 | JAPAN SOCIETY FOR THE PROMOTION OF SCIENCE | 18 |
| 7 | GRANTS IN AID FOR SCIENTIFIC RESEARCH KAKENHI | 17 |
| 8 | ASSOCIATION FRANCAISE CONTRE LES MYOPATHIES | 15 |
| 9 | NIH NATIONAL INSTITUTE OF GENERAL MEDICAL SCIENCES NIGMS | 14 |
| 10 | NIH NATIONAL INSTITUTE OF ARTHRITIS MUSCULOSKELETAL SKIN DISEASES NIAMS | 10 |
| 11 | UK RESEARCH INNOVATION UKRI | 9 |
| 12 | GERMAN RESEARCH FOUNDATION DFG | 8 |
| 13 | MUSCULAR DYSTROPHY ASSOCIATION | 8 |
| 14 | CANADIAN INSTITUTES OF HEALTH RESEARCH CIHR | 7 |
| 15 | EUROPEAN UNION EU | 7 |
| 16 | FUNDACAO DE AMPARO A PESQUISA DO ESTADO DE SAO PAULO FAPESP | 7 |
| 17 | MEDICAL RESEARCH COUNCIL UK MRC | 7 |
| 18 | NIH NATIONAL INSTITUTE OF NEUROLOGICAL DISORDERS STROKE NINDS | 7 |
| 19 | MINISTRY OF EDUCATION UNIVERSITIES AND RESEARCH MIUR | 6 |
| 20 | NATURAL SCIENCES AND ENGINEERING RESEARCH COUNCIL OF CANADA NSERC | 6 |
