# Supplementary material for: Global MyoG research 2004–2024: a bibliometric analysis of trends and translational implications
Source: Exp Biol Med (Maywood). 2026 Mar 5;251:10929. doi: 10.3389/ebm.2026.10929 (PMC12999542; doi:10.3389/ebm.2026.10929)
Supplement: Supplementary file 6 [file Table4.docx]

**Supplementary File 4.** Annual publication volume and related bibliometric indices in MyoG research (2004–2024).

| **Rank** | **Journals** | **H-index** | **G-index** | **Count** | **Citations** | **IF2023** |
| --- | --- | --- | --- | --- | --- | --- |
| 1 | JOURNAL OF BIOLOGICAL CHEMISTRY | 15 | 18 | 18 | 844 | 4 |
| 2 | PLOS ONE | 12 | 14 | 14 | 207 | 2.9 |
| 3 | BIOCHEMICAL AND BIOPHYSICAL RESEARCH COMMUNICATIONS | 6 | 9 | 9 | 170 | 2.5 |
| 4 | JOURNAL OF CELL SCIENCE | 6 | 6 | 6 | 136 | 3.3 |
| 5 | MOLECULAR AND CELLULAR BIOLOGY | 6 | 6 | 6 | 543 | 3.2 |
| 6 | SCIENTIFIC REPORTS | 6 | 6 | 6 | 71 | 3.8 |
| 7 | GENETICS AND MOLECULAR RESEARCH | 5 | 7 | 8 | 22 | 0.6 |
| 8 | JOURNAL OF CELLULAR PHYSIOLOGY | 5 | 6 | 6 | 406 | 4.5 |
| 9 | AMERICAN JOURNAL OF PHYSIOLOGY-CELL PHYSIOLOGY | 4 | 4 | 4 | 126 | 5 |
| 10 | CELL AND TISSUE RESEARCH | 4 | 4 | 4 | 678 | 3.2 |
