# Supplementary material for: Global MyoG research 2004–2024: a bibliometric analysis of trends and translational implications
Source: Exp Biol Med (Maywood). 2026 Mar 5;251:10929. doi: 10.3389/ebm.2026.10929 (PMC12999542; doi:10.3389/ebm.2026.10929)
Supplement: Supplementary file 7 [file Table6.docx]

**Supplementary File 6.** Top 10 MyoG-related papers ranked by normalised citation counts.

| **RANK** | **Title** | **Doi** | **Year** | **Normalized TC** | **Total Citations** |
| --- | --- | --- | --- | --- | --- |
| 1 | Myogenic factors that regulate expression of muscle-specific microRNAs | 10.1073/pnas.0602831103 | 2006 | 8.67 | 573 |
| 2 | Myogenin is an essential regulator of adult myofibre growth and muscle stem cell homeostasis. | 10.7554/eLife.60445 | 2020 | 7.34 | 89 |
| 3 | An Origanum majorana Leaf Diet Influences Myogenin Gene Expression, Performance, and Carcass Characteristics in Lambs. | 10.3390/ani13010014 | 2023 | 7.02 | 29 |
| 4 | Reciprocal inhibition between Pax7 and muscle regulatory factors modulates myogenic cell fate determination. | 10.1083/jcb.200608122 | 2007 | 5.88 | 231 |
| 5 | Low-load high volume resistance exercise stimulates muscle protein synthesis more than high-load low volume resistance exercise in young men. | 10.1371/journal.pone.0012033 | 2010 | 5.83 | 386 |
| 6 | Myogenin and class II HDACs control neurogenic muscle atrophy by inducing E3 ubiquitin ligases. | 10.1016/j.cell.2010.09.004 | 2010 | 5.39 | 357 |
| 7 | Myomaker, Regulated by MYOD, MYOG and miR-140-3p, Promotes Chicken Myoblast Fusion. | 10.3390/ijms161125946 | 2015 | 5.13 | 89 |
| 8 | An initial blueprint for myogenic differentiation. | 10.1101/gad.1281105 | 2005 | 4.51 | 366 |
| 9 | Insulin-like growth factor-1 receptor is regulated by microRNA-133 during skeletal myogenesis. | 10.1371/journal.pone.0029173 | 2011 | 4.02 | 125 |
| 10 | Genetic background and embryonic temperature affect DNA methylation and expression of myogenin and muscle development in Atlantic salmon (Salmo salar). | 10.1371/journal.pone.0179918 | 2017 | 3.85 | 62 |
